# ModiCal: A Targeted Calibration Workflow for Site-Specific m^5^C Validation by Nanopore Direct RNA Sequencing

**DOI:** 10.1021/acschembio.6c00009

**Published:** 2026-05-19

**Authors:** Zeynep Özrendeci, Stefan Mündnich, Stefan Pastore, Chia Ching Wu, Virginie Marchand, Yuri Motorin, Alessia Ruggieri, Susanne Gerber, Mark Helm

**Affiliations:** † Institute of Pharmaceutical and Biomedical Science (IPBS), 9182 Johannes Gutenberg University, 55128 Mainz, Germany; ‡ Department of Infectious Diseases, Molecular Virology, Center for Integrative Infectious Disease Research, Medical Faculty Heidelberg, Heidelberg University, 69120 Heidelberg, Germany; § EpiRNA-Seq Core Facility, Université de Lorraine, SMP IBSLor, F-54000 Nancy, France; ∥ Université de Lorraine, CNRS, UMR7365 IMoPA, F-54000 Nancy, France; ⊥ Institute of Human Genetics, University Medical Center, Johannes Gutenberg University Mainz, 55131 Mainz, Germany; # Institute for Quantitative and Computer Biosciences (IQCB), 55128 Mainz, Germany

## Abstract

Accurate identification of RNA 5-methylcytidine (m^5^C) at the single-nucleotide resolution remains a central challenge in nanopore direct RNA sequencing (DRS). Current global scanning and modification-aware basecalling methods enable transcriptome-wide profiling but often yield high false-positive rates and lack site-specific accuracy. To address this, we repurposed ModiDeC, originally a de novo multimodification classifier, into a targeted, high-precision validation tool for RNA modification sites with prior biochemical knowledge. This was implemented through a three-step calibration workflow that alternates between biochemical and computational modules using the well-characterized m^5^C2278 site in 25S rRNA as a starting point. Baseline training uses short synthetic RNAs carrying either a methylated or unmodified C2278 as ground truth, followed by IVT-derived calibration and validation in methyltransferase knockout yeast. The baseline model accurately detected the bona fide m^5^C2278 site but initially produced off-target predictions. Iterative retraining with unmodified IVT signals progressively reduced and ultimately eliminated false positives while maintaining a strong signal at the bona fide site. The final model retained enzyme-dependent detection in wild-type versus knockout yeast and, when explicitly targeted, was also able to detect the second rRNA site, C2870, which remained invisible in the initial analysis. Application to native human prerRNA processing intermediates further resolved two distinct m^5^C deposition regimes on 28S rRNA, while generalization to dengue virus genomic RNA confirmed that the same calibration logic transfers across diverse RNA contexts. Together, this study establishes a reproducible and transferable framework that integrates biochemical validation with iterative neural network refinement, providing a route toward reliable site-specific m^5^C confirmation by nanopore direct RNA sequencing.

## Graphical abstract

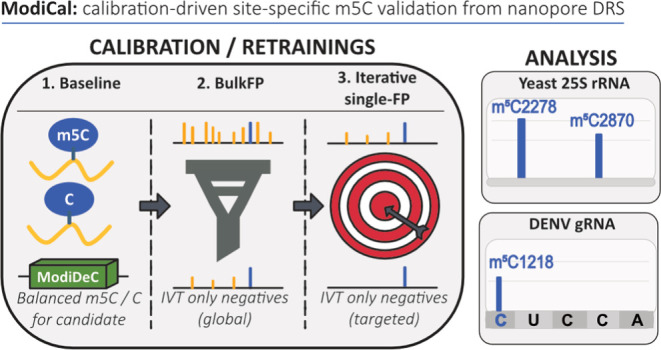

## 1 Introduction

RNA modifications add an additional regulatory layer beyond the canonical nucleotide sequence, influencing RNA metabolism, stability, and translation.^1,2^ Among these, m^5^C is one of the most widespread modifications.^3^ It occurs across multiple RNA classes, including rRNA, tRNA,^4,5^ mRNA,^6,7^ and various noncoding RNAs, where it modulates RNA folding, stability, and interaction with effector proteins.^8^ Accumulating evidence links aberrant m^5^C deposition or reader protein function to diverse cellular processes and disease contexts, including tumorigenesis in bladder, liver, brain, and hematopoietic systems.^9–12^ Despite its biological importance, m^5^C stoichiometry across RNA species remains debated, largely due to the lack of detection approaches that are both sensitive and quantitatively reliable.

Among current detection strategies, bisulfite sequencing remains the benchmark for nucleotide-resolution m^5^C mapping.^13–15^ It enables quantification of modification stoichiometry; however, it is limited by two opposing challenges: incomplete C-to-U conversion during bisulfite treatment, to which m^5^C is resistant, and extensive RNA degradation. The harsh chemical conditions required for full conversion often fragment RNA, whereas milder treatments preserve RNA integrity but leave unconverted cytidines that appear as false positives (FP).^16^ These competing reactions, inherent to bisulfite chemistry, create a trade-off between sensitivity and specificity that hampers reliable m^5^C mapping and quantification. Optimized variants such as Ultrafast Bisulfite Sequencing (UBS-seq) improve conversion efficiency, reduce nucleic-acid degradation, and enable more quantitative detection of m^5^C. Nevertheless, UBS-seq still relies on C-to-U conversion, which increases the resulting sequence similarity and thus hampers accurate alignment, and it cannot distinguish m^5^C from hm^5^C.^16^

Alternatively, antibody-based enrichment methods, including m^5^C-RNA immunoprecipitation (RIP)-seq^17^ and 5-azacytidine-mediated RIP,^18^ depend on antibody specificity, lack single-nucleotide resolution, and cannot quantify site-specific m^5^C stoichiometry. Cross-linking approaches such as miCLIP depend on enzyme variants, which limit their applicability and often fail to detect m^5^C sites in highly structured RNAs.^19^

To overcome these limitations, third-generation long-read technologies now enable direct interrogation of RNA modifications on native molecules. Oxford Nanopore direct RNA sequencing (DRS) simultaneously provides sequence and chemical information without reverse transcription or amplification. Modified nucleotides perturb the ionic current as RNA translocates through a nanopore, generating characteristic signal deviations that can be exploited to infer modification presence and frequency.^20,21^

More than 20 computational frameworks have been developed to extract modification information from DRS data, which can be divided into three main categories:^22^

(1) Error-based methods, identifying modified sites from systematic basecalling errors such as mismatches, insertions, or deletions (e.g., ELIGOS,^23^ DRUMMER,^24^ EpiNano,^25^ and JACUSA2 ^26^);
(2) Signal-based methods, which quantify raw current features such as mean current, dwell time, and trace shape (e.g., MINES,^27^ nano-ID,^28^ nanoRMS,^21^ Nanocompore,^29^ NanoPsu,^30^ xPore,^31^ nanoDoc2,^32^ Nanom6A,^33^ m6Anet,^34^ DENA,^35^ Penguin,^36^ CHEUI,^37^ mAFiA,^38^ NanoSPA,^39^ TandemMod,^40^ SingleMod,^41^ ModiDeC,^42^ DirectRM^43^); and
(3) Modification-aware basecallers, which directly integrate modification prediction into basecalling via neural networks trained on labeled signal data (e.g., IL-AD,^44^ m6ABasecaller,^45^ Dorado (ONT)).

Although widely used, error-based approaches often confuse structural effects with true modification signals, leading to false positive assignments. In addition, nanopore current is shaped by several nucleotides occupying the pore at the same time such that the measured signal reflects k-mer context rather than individual bases. Comparative approaches relying on matched wild-type and modification-depleted samples are limited by the need for biologically paired conditions, which are not always available or practical.^43,46^ As a more recent alternative, Oxford Nanopore Technologies (ONT) introduced Dorado, a modification-aware basecaller supporting pseudouridine, m6A, inosine, and m^5^C detection, currently representing the only available m^5^C caller compatible with the RNA004 chemistry. However, independent benchmarking studies have revealed high false-positive rates across modification types, with up to 40% of sites detected at higher modification frequency in unmodified IVT controls than in wild-type samples.^47^ While this trend was observed for all modifications, performance was particularly poor for m^5^C. In *Escherichia coli*, for instance, only one of three known rRNA m^5^C sites was detected, leading the authors to discontinue downstream analyses using the Dorado m^5^C model due to its weak signal separation between wild-type and IVT controls.^47^

Beyond limitations specific to individual tools, m^5^C itself remains intrinsically difficult to detect by nanopore DRS. Its low abundance and the strong influence of neighboring nucleotides on signal profiles hinder reliable discrimination of genuine modification signals from background variation. Additional challenges arise from species-specific signal variation and the 3′ coverage bias characteristic of DRS.^48–50^ Consistent with this, the developers of CHEUI reported a trade-off between recall and false-positive rate, weak overlap with bisulfite-based data sets, and limited signal separation between modified and unmodified reads.^38^ Moreover, a recent large-scale comparative evaluation of nanopore-based RNA modification detection tools identified m^5^C as one of the poorest-performing modifications across multiple algorithms and both RNA002 and RNA004 chemistries, even when models were retrained.^51^

To address this gap, we repurposed the ModiDeC neural network, originally developed as a multimodification classifier, within a calibration-driven strategy for targeted, site-specific validation of RNA modification sites, which we term ModiCal. We first established and refined this calibration workflow using the well-characterized m^5^C2278 site in *Saccharomyces cerevisiae* 25S rRNA, where an initial model was trained on synthetic ground truth, followed by iterative retraining with unmodified IVT reference signals specifically selected from false-positive positions to suppress background while stabilizing detection of the true site. Building on this framework, we then extended the calibration to include the second known rRNA m^5^C site, C2870, yielding a benchmark model that accurately recovers both bona fide sites with their enzyme-dependent behavior in wild-type and knockout yeast samples. Extending the framework to a mammalian system, we profiled the two conserved m^5^C sites of human 28S rRNA across the prerRNA processing cascade and resolved two distinct maturation-dependent deposition regimes, a near-stoichiometric m^5^C4447 already present on the earliest 47S primary transcript and a progressively installed m^5^C3782 that tracks late 28S maturation, demonstrating that ModiCal can deliver site-specific biological insight from native RNA. Together with further generalization to the dengue virus genomic RNA (Supporting Information), ModiCal provides a reproducible calibration framework for high-precision, site-specific m^5^C validation by nanopore DRS.

## 2 Results

### 2.1 Baseline Performance of a Ground-Truth-Trained m^5^C Model

We first tested whether a model trained on paired modified and unmodified signal chunks as “ground truth” from a defined sequence context can recover the true site in full-length native RNA while avoiding false positives. We chose the well-characterized, highly modified m^5^C2278 site as the initial target.^52–55^ A second reported site in the same rRNA, m^5^C2870, was not included at this stage and was reserved as an internal benchmark to assess how well the calibration generalizes beyond the initial context.

To generate ground truth, we designed synthetic 99 nt constructs that precisely mimicked the native *S. cerevisiae* 25S rRNA sequence context surrounding position 2278, carrying either a modified or unmodified cytidine at position 50 (corresponding to position 2278 in the native RNA). The constructs were synthesized via splint ligation, in which three oligoribonucleotides are brought into the correct orientation by a complementary DNA splint. The central oligo contained either a methylated or canonical cytidine, resulting in a point-modified synthetic RNA upon ligation (Figure 1).^56^

**Figure 1:**
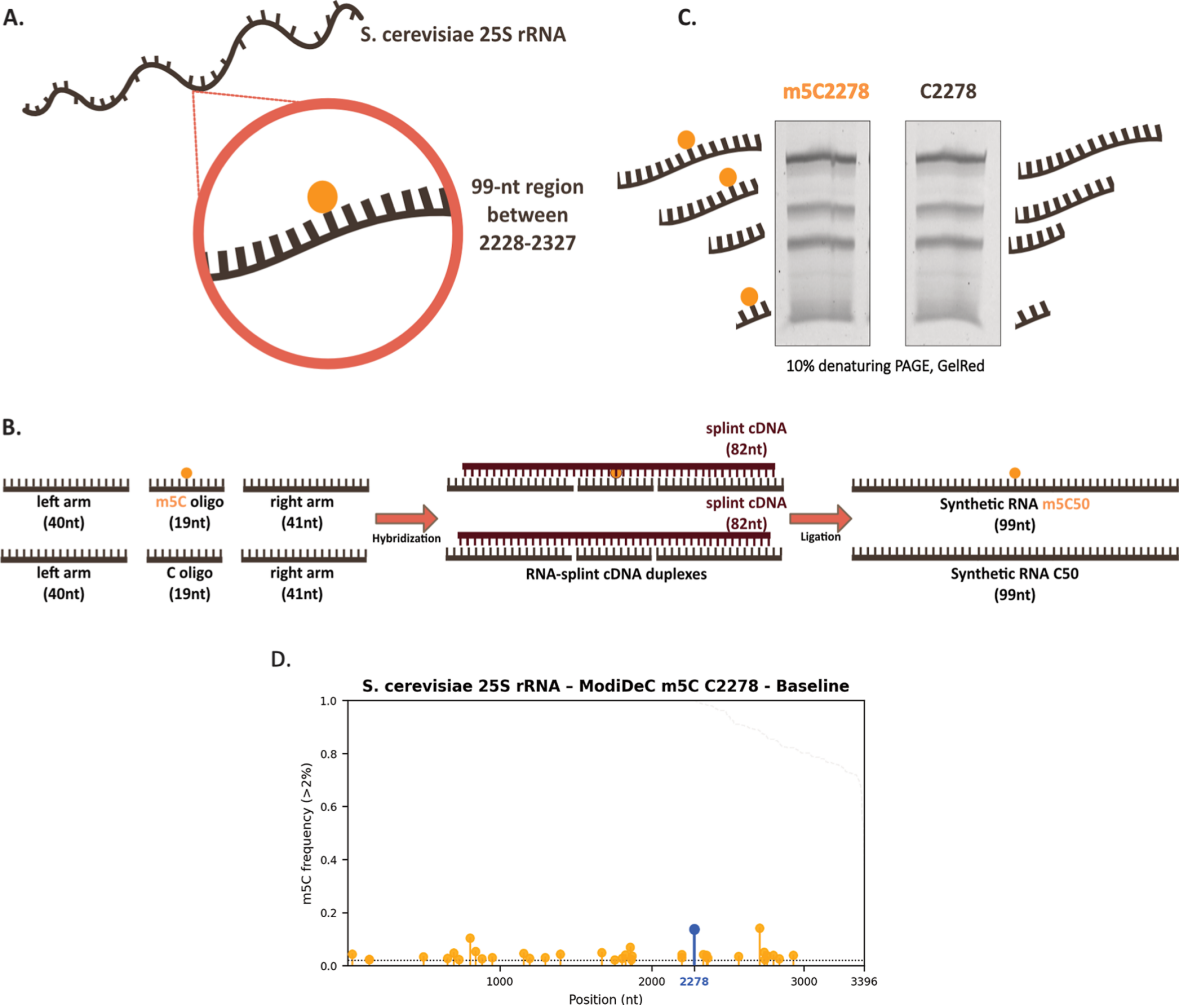
Synthetic ground-truth RNAs used for baseline training. (A) Schematic of the *S. cerevisiae* 25S rRNA region used for ground-truth generation (positions 2228–2327), containing the native m5C2278 site. (B) Splint-ligation strategy for ground truth generation. (C) Denaturing PAGE (10%) showing ligated m5C-modified and unmodified RNA constructs. (D) Baseline ModiDeC m5C prediction profile across native *S. cerevisiae* 25S rRNA.

Synthetic modified and unmodified constructs were sequenced by Nanopore DRS, each yielding >10 million reads; only 100,000 reads (∼1%) were used for signal-chunk curation. Following curation (Methods), normalized current and dwell-time features, together with one-hot encoded reference sequence, were packaged into .npz files (multiarray containers). With 512 signal chunks per .npz, these 100,000 reads produced >1000 training files, forming the ground-truth data sets used to train the Baseline Model.

Before baseline training, we tested how data set size affects learning and accuracy by training models with increasing numbers of curated chunks (200–1600.npz per class) while keeping hyperparameters constant. Performance improved up to 800.npz per class, which gave the fastest, most stable convergence and the best-balanced metrics, whereas larger sets provided no additional gain (Figure S1). We therefore used 800.npz per class for all subsequent baseline training. Applying this Baseline model to native 25S rRNA using a publicly available yeast total RNA Nanopore DRS data set,^57^ the model detected the true m^5^C2278 site, but with a lower frequency (<20%) (Figure 1D) than reported previously.^52^ It also misclassified 35 additional sites as m^5^C, with some reaching frequencies comparable to the true-positive signal.

Notably, the second reported m^5^C site on 25S rRNA, m^5^C2870, was not detected by the Baseline model at this stage, suggesting that the representation learned from the C2278 training set does not automatically generalize to other conserved sites. This observation raises the question of whether additional prior knowledge or targeted calibration is required for recognizing m^5^C2870, an issue that we address later in this study.

These findings demonstrate that while local training enables recognition of the targeted modification site, it does not on its own ensure accurate stoichiometry estimates or prevent widespread FP, motivating the need for further calibration steps before additional sites can be meaningfully assessed.

### 2.2 Iterative Calibration Eliminates False Positives and Recovers Accurate m^5^C Stoichiometry

We next asked whether targeted retraining on previously misclassified sites could reduce FP calls without impairing the detection of the true modification.

Initially, retraining on balanced modified and unmodified signal chunks from the most recurrent FP site failed to alter its predicted frequency, indicating that balanced input alone was insufficient to correct this misclassification. Because a FP call reflects a bias toward “modified” classification where the site is actually unmodified, we hypothesized that adding only unmodified examples would restore balance. The model was therefore retrained by supplementing the baseline training pool exclusively with unmodified signal chunks and the yeast 25S rRNA data set was reanalyzed. This strategy successfully removed the targeted FP site while leaving other predictions unchanged, indicating that each FP site must be targeted by incorporating its unmodified signals into the training data set.

Since generating splint-ligated constructs for all 35 FP sites would be costly and laborious, we opted to produce full-length IVT 25S rRNA as a comprehensive unmodified reference. Nanopore DRS of the IVT yielded ∼200,000 reads. Data curation targeting all FP positions using 5000 reads produced ∼700.npz files ready for retraining. Because unmodified signals from all FP positions were introduced simultaneously, i.e., in bulk, this step was termed the Bulk False Positive (BulkFP) model.

At the global level, bulkFP retraining preserved overall classification performance (Figure 2A,D). When applied to native *S. cerevisiae* 25S rRNA (Figure S2A), the BulkFP model achieved a >90% reduction in FPs, while the true m^5^C2278 signal increased to 76%. Together, these results demonstrate that BulkFP suppression provides an efficient, interpretable calibration mechanism that strongly reduces background while preserving true-site sensitivity. Despite this improvement, a small number of FPs persisted. Because IVT-derived signals had been added in bulk, some FP sites may be under-represented due to uneven transcript coverage. We therefore asked whether the remaining background can be fully cleared through iterative site-specific calibration.

**Figure 2:**
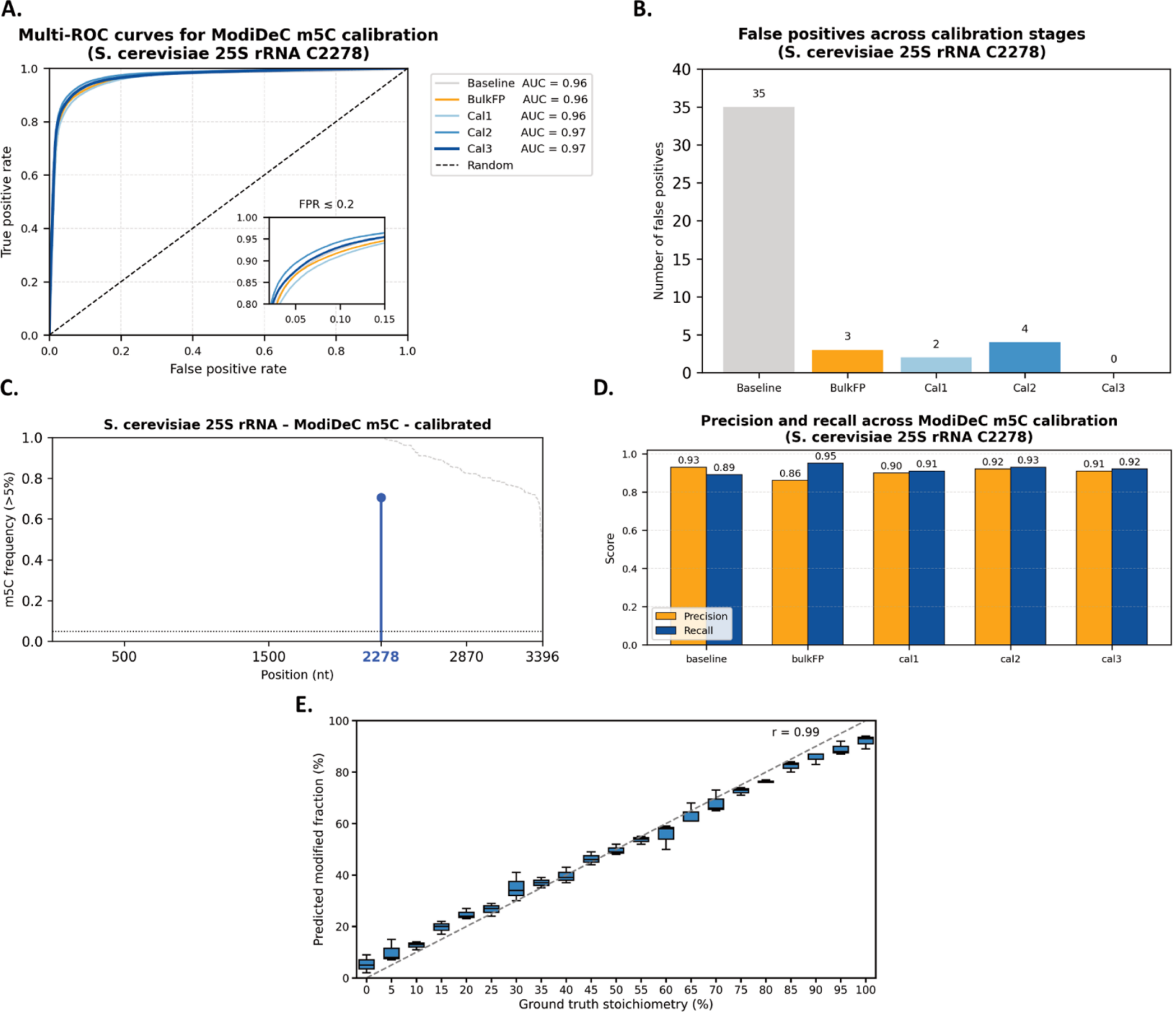
Bulk and iterative calibration progressively eliminate FP and recover accurate m5C stoichiometry. (A) ROC curves comparing the baseline model, the BulkFP retraining step, and three iterative calibration rounds (Cal1–Cal3). (B) Number of FP sites detected in native yeast 25S rRNA across calibration stages. (C) 25S rRNA m5C2278 prediction profiles after full calibration. (D) Precision and recall across calibration rounds. (E) Synthetic titration experiment using splint-ligated RNAs containing defined fractions of m5C2278 reads.

To address this, we implemented an iterative single-FP training strategy to further refine model specificity. Unlike the BulkFP step, which incorporated unmodified IVT signals from all FP positions simultaneously, this stage targeted each remaining FP site individually, ensuring that every FP position was explicitly represented. To assess the minimal amount of training data required, only one. npz file per FP site was added to maintain data set balance. Complete background suppression was achieved after three calibration rounds (Cal1–Cal3), while the true modification site m^5^C2278 remained stably detected at comparable frequencies throughout (Figure S2B–D). Reanalysis using the final calibrated model (Cal3) (Figure 2C) showed a single peak at C2278 with ∼71% predicted m^5^C frequency, while all other positions stayed below the 5% threshold, in good agreement with previous bisulfite sequencing.^52^

Across calibration rounds, ROC curves converge toward a stable discriminative profile, indicating progressive suppression of misclassification noise (Figure 2A). The FP count plot (Figure 2B) highlights the practical outcome of this trend, showing a stepwise reduction from widespread overprediction to a clean map without detectable background. The precision/recall bar plot (Figure 2D) summarizes how sensitivity and specificity coevolve, with both metrics converging around 0.9–0.93 from Cal1 onward.

To independently confirm that calibration preserves quantitative accuracy, we performed a synthetic titration using splint-ligated constructs with defined m^5^C2278 stoichiometries (0–100%). Predicted modification fractions closely matched the designed input mixtures (*r* = 0.99; Figure 2E), demonstrating that the calibrated model provides quantitatively reliable estimates at the true-positive site while suppressing off-target signal elsewhere.

Overall, iterative addition of empirically defined unmodified signals, first in bulk and then site-by-site, drives progressive rebalancing of the ModiDeC network, transforming a broadly trained baseline classifier into a high-precision, site-specific detector for m^5^C2278 in yeast 25S rRNA. Since the calibrated model remained specific to m^5^C2278, the next question was whether incorporating m^5^C2870 into the calibration process would allow the network to learn this second conserved site and, ultimately, detect both bona fide sites in biological samples. We examine this in the following section.

### 2.3 Dual-Site Calibration Yields Enzyme-Dependent m^5^C Detection of Yeast 25S rRNA

In *S. cerevisiae*, the large-subunit rRNA contains exactly two conserved m^5^C residues, C2278 and C2870, which are installed by distinct NOP2/Sun family methyltransferases: Rcm1 (NSUN5) modifies C2278, whereas Nop2 (NSUN1) methylates C2870 (Table S1).^52–55,58,59^ Because calibration on m^5^C2278 alone did not generalize to m^5^C2870, we asked whether explicitly incorporating this second site into training would allow the network to learn both modifications. Previous studies consistently report near-stoichiometric modification of both sites in wild-type cells, with selective loss of C2278 in Rcm1 knockout (rcm1Δ) and of C2870 in Nop2 knockout (nop2Δ), providing a stringent benchmark for testing enzyme-dependent, dual-site resolution in biological samples.

As an independent biochemical reference, we analyzed RNA from wild-type (WT), rcm1Δ, and nop2Δ yeast strains by bisulfite sequencing (Figure S3). Consistent with previous reports,^52–55^ WT 25S rRNA showed two bisulfite-resistant peaks at C2278 and C2870, with selective loss of C2278 in rcm1Δ and loss of C2870 in nop2Δ, while all other cytidines remained at baseline levels. These data confirm that C2278 and C2870 are the only detectable m^5^C sites on 25S rRNA under our conditions and validate their expected enzyme dependence.

To validate these findings by nanopore DRS, we sequenced total RNA from WT, rcm1Δ, and nop2Δ cells (two biological replicates each), yielding ∼7 million reads per sample and ∼300,000 aligned 25S rRNA reads in total. To extend the single-site model to both known 25S rRNA m^5^C positions, we curated an additional training set for C2870 directly from native reads, using WT signals as the modified class and nop2Δ signals as the unmodified class, and added these in balanced numbers to the existing C2278-based training pool. Consequently, the network learned C2278 from synthetic ground truth and C2870 from native genetic controls. The expanded model was calibrated using the same ModiCal strategy, and all 25S rRNA profiles in Figure 3A were generated from the analysis of nonoverlapping WT, rcm1Δ, and nop2Δ read sets, ensuring that m^5^C2870 detection reflects true modification rather than reuse of training data.

**Figure 3:**
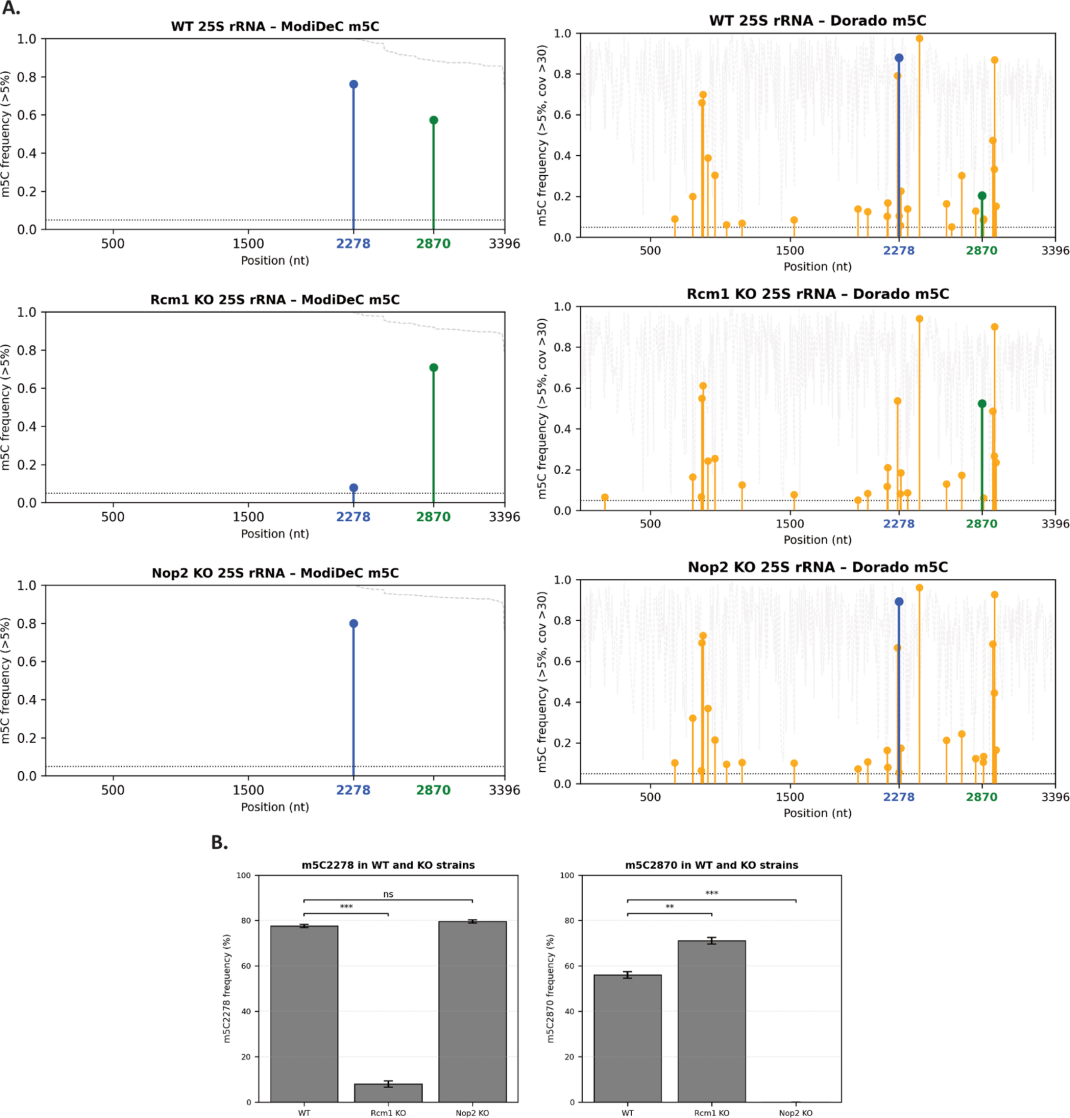
Validation of calibrated m5C model in wild-type and knockout yeast strains. (A) m5C profiles across yeast 25S rRNA obtained with the calibrated ModiDeC model (left) and Dorado m5C (right) for WT, Rcm1 KO, and Nop2 KO samples. C2278 (blue), C2870 (green), off-target calls (orange). (B) Quantification of m5C2278 and m5C2870 frequencies in WT, Rcm1 KO, and Nop2 KO samples using the calibrated model.

With the calibrated model, 25S rRNA profiles were clean and readily interpretable (Figure 3A). In WT cells, two clear m^5^C peaks were detected at C2278 (∼76–78%) and C2870 (∼55–57%). In rcm1Δ, the C2278 signal dropped to baseline while C2870 remained above threshold and showed a modest increase (∼70–72%). Conversely, in nop2Δ, the C2870 peak was selectively lost whereas C2278 was retained at WT-like levels (∼78–80%). These enzyme-dependent patterns were consistent across biological replicates and are summarized quantitatively in Figure 3B, confirming selective loss of m^5^C2278 in rcm1Δ and of m^5^C2870 in nop2Δ.

Apart from C2278 and C2870, no other cytidines in 25S rRNA exceeded the detection threshold in any strain/replicate when analyzed with the calibrated ModiDeC m^5^C model. Shown in Figure 3A (right), Dorado analysis of the same reads correctly detected the true-positive sites with their expected enzyme dependence; however, it produced a substantially more complex landscape, reporting ∼25 additional high-confidence m^5^C calls across 25S rRNA, including several sites consistently called above 80% in all samples despite lacking biochemical support. Notably, the commonly used stringent cutoff (0.95) filters out many reads, reducing per-position coverage (light gray trace) and making coverage more uneven; lowering the cutoff to 0.50 restores coverage but increases false positives from ∼25 to ∼160 sites on average (Figure S4). Together, these results demonstrate that the calibrated ModiDeC m^5^C model acts as a truly site-specific validation tool on yeast 25S rRNA: it accurately recovers the two known m^5^C sites with their correct enzyme dependencies while suppressing spurious calls, thereby establishing a robust foundation for testing transferability to more complex RNA substrates.

### 2.4 ModiCal Reveals Two Distinct m^5^C Deposition Regimes across Human prerRNA Processing Intermediates

To test whether ModiCal can yield new biological insight in a mammalian system, we applied it to the two conserved m^5^C sites of human 28S rRNA, m^5^C3782, and m^5^C4447, which are the direct orthologues of yeast m^5^C2278 and m^5^C2870 validated in Section 3. A dedicated dual-site ModiCal model was trained for human 28S rRNA from synthetic and native signal chunks, providing matched modified and unmodified references, following the same workflow used for yeast.

To resolve whether m^5^C deposition at these two sites is temporally coordinated or independent during ribosome biogenesis, we analyzed nine pre-rRNA species spanning the full 28S maturation cascade, from the 47S primary transcript through the 45S, 43S, 41S, 36S, 36S-C, 32S, and 28.5S precursors to the mature cytoplasmic 28S. Intermediate pre-rRNAs had been isolated from nucleolar (SN3) total RNA using the Nanoribolyzer workflow and profiled by nanopore DRS in three independent biological replicates in a previous study;^60^ here, we reanalyzed these data sets using ModiCal. Applying the calibrated dual-site model produced reproducible, enzyme-specific m^5^C profiles across the entire maturation cascade (Figure 4A). m^5^C4447 was already at 0.80 ± 0.05 on the 47S primary transcript, plateaued near-stoichiometrically by the 45S intermediate (0.86 ± 0.03), and remained between 0.83 and 0.88 across every downstream intermediate, reaching 0.88 ± 0.01 on mature 28S. In contrast, m^5^C3782 started at 0.65 ± 0.03 on 47S and climbed progressively through the processing cascade to 0.88 ± 0.02 on mature 28S. The maturation-dependent m^5^C gain differed markedly between them, with an increase of 0.23 for C3782 compared with only 0.08 for C4447 between the 47S transcript and the mature 28S.

**Figure 4:**
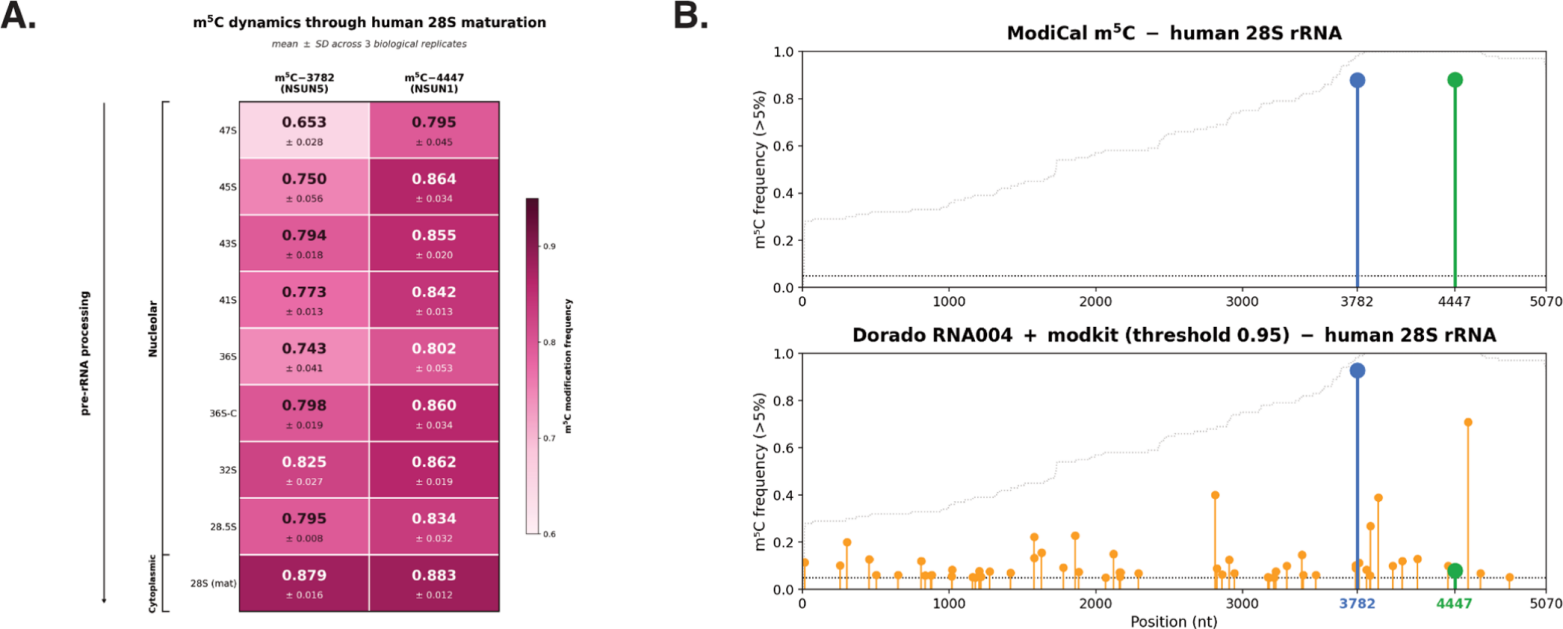
ModiCal resolves two distinct m^5^C deposition regimes across human pre-rRNA processing. (A) Heatmap of mean ± SD m^5^C modification frequency at C3782 and C4447 across the 47S primary transcript, seven nucleolar precursors (45S, 43S, 41S, 36S, 36S-C, 32S, 28.5S) and the mature cytoplasmic 28S, from SN3 total RNA (*n* = 3 biological replicates; 47S replicate 2 excluded due to low read count). (B) Position-resolved m^5^C calls on mature 28S reads by calibrated ModiCal (top) and by Dorado m^5^C and Modkit (threshold 0.95, bottom). True-positive sites C3782 (blue) and C4447 (green); orange, off-target calls. Gray trace, cumulative coverage.

These two regimes are consistent with the known biology of the responsible enzymes. NSUN1 is a stable component of the human UTP-B subcomplex of 90S assembly factors and directly binds the 5′ETS of the 47S primary transcript,^61^ providing a structural basis for the near-complete deposition of m^5^C4447 already on 47S. m^5^C3782, in contrast, lies in the domain IV peptidyl-transferase neighborhood that is progressively restructured during late pre-60S maturation, and its gradual accumulation mirrors the late-deposition pattern established by kinetic labeling of 25S 2′-*O*-methylations.^58^

Importantly, Dorado m^5^C analysis of the same mature 28S reads detected m^5^C3782 but failed to call m^5^C4447 (false negative) and produced 57 additional high-confidence off-target calls across 28S (Figure 4B), whereas calibrated ModiCal recovered both bona fide sites with zero off-target calls. Together, these results identify two temporally distinct m^5^C regimes on a single rRNA molecule, an early, near-stoichiometric m^5^C4447 plateaued by the earliest post-47S intermediate and a progressively installed m^5^C3782 that tracks late 60S maturation, providing, to our knowledge, the first per-intermediate quantitative view of m^5^C deposition kinetics on native prerRNA.

## 3 Discussion

A central message of this work is that ModiCal does not introduce a new prediction tool but demonstrates the value of systematically interrogating an existing, retrainable neural network before developing task-specific models. Neural networks are often treated as black boxes whose behavior is assumed to be fixed once trained. By analyzing how a ModiDeC-based model responds to different training regimes, FP patterns, and calibration inputs, we show that model behavior can be actively shaped and repurposed through targeted retraining. This illustrates a broader principle: careful calibration of a flexible network can uncover application-specific capabilities without changing the underlying architecture.

Conceptually, the iterative retraining procedure underlying ModiCal operates as a form of hard-negative mining applied to a nanopore-signal classifier: at each calibration round, the IVT positions on which the current model places its most confident FP calls are extracted as negatives and reintroduced into training, progressively pushing the decision boundary away from canonical-cytidine signals that most closely resemble the modified class. Hard-negative mining has been central to training high-precision deep classifiers in computer vision and embedding learning,^62,63^ and its translation here into nanopore DRS provides a principled explanation for why BulkFP and iterative single-FP rounds progressively collapse transcript-wide FPs while preserving signal at the bona fide modified site. In this framing, FPs are not noise to be suppressed with ad hoc thresholds but diagnostic readouts where decision boundaries are misaligned, and the choice of training inputs is itself the calibration lever. Sites exposed only to an unmodified IVT signal are consistently pushed toward the negative class, whereas sites trained with balanced modified and unmodified examples retain strong, enzyme-dependent contrast between conditions. This same logic explains why calibration centered on a single site may not generalize to additional modification sites on the same RNA; recovery of further sites (as exemplified by m^5^C2870) requires explicit inclusion of balanced, site-specific training data rather than implicit generalization.

More broadly, this work informs the debate on synthetic versus native training data. Synthetic-only training can overinterpret complex native backgrounds, whereas native-only training is constrained by motif availability, enzyme specificity, and partial modification state. ModiCal shows that synthetic and native ground truths can be combined effectively within one calibration workflow when training inputs are integrated in a controlled, context-aware manner.

Extending ModiCal to human prerRNA processing intermediates achieved two things that a yeast rRNA or a viral genome alone could not have delivered. First, it showed that the same three-step calibration transfers across a eukaryotic rRNA, a viral genomic RNA (Figure S5), and a mammalian rRNA without architectural change, consolidating site-specific calibration as a substrate-agnostic layer. Second, it uncovered that the two conserved m^5^C sites of human 28S rRNA follow independent maturation regimes on the same RNA molecule, a kinetic separation that transcriptome-wide callers obscured: on the same reads, Dorado m^5^C recovered m^5^C3782 but did not report m^5^C4447 at its standard threshold, consistent with sequence-context-dependent blind spots inherent to general-purpose basecalling. On this basis, ModiCal currently provides, to our knowledge, the only high-precision, stoichiometric route from RNA004 nanopore reads to per-site m^5^C quantification in native samples.

Finally, ModiCal operates as a prior-knowledge-dependent validator, and here we demonstrate background-free behavior on RNAs up to ∼10 kb. We therefore propose its use in a two-tier “map-and-validate” strategy: transcriptome-wide de novo callers nominate candidate sites, and calibration-based validation is applied to a selected subset where high-confidence, site-specific evidence is required.

## 4 Methods

Complete experimental details are provided in the Supporting Information.

### 4.1 RNA Preparation and Synthetic Training Constructs

Full-length *S. cerevisiae* 25S rRNA was generated by IVT from a linearized pUC57 plasmid (GenScript, USA) carrying the complete 25S rRNA sequence under control of a T7 promoter. Plasmids were linearized with *Bam*HI (New England Biolabs, Germany). IVT was performed using the HiScribe T7 High Yield RNA Synthesis Kit (New England Biolabs, Germany) following the manufacturer’s protocol. DNA templates were removed by DNase I digestion (Thermo Fisher Scientific, Germany), and RNA was purified using the Monarch RNA Cleanup Kit (New England Biolabs, Germany). RNA integrity and yield were assessed by agarose gel electrophoresis and NanoDrop spectrophotometry (Thermo Fisher Scientific).

Synthetic ground-truth RNAs for model training were generated by splint ligation. For yeast C2278 and DENV C1218, chemically synthesized RNA oligonucleotides were assembled into sequence-identical constructs carrying either methylated or canonical cytidine at the target position. Yeast m^5^C-containing oligonucleotides were obtained from Dharmacon (Germany), whereas unmodified and flanking oligonucleotides were purchased from Biomers (Germany). Oligonucleotides were phosphorylated using T4 polynucleotide kinase (Thermo Fisher Scientific), annealed to a complementary DNA splint, and ligated using T4 DNA ligase (Thermo Fisher Scientific). Ligated products were purified by denaturing polyacrylamide gel electrophoresis and quantified prior to sequencing.

DENV IVT RNA and native gRNA were generated or purified as described previously.^64^

### 4.2 Polyadenylation and Nanopore Direct RNA Sequencing

Synthetic RNAs and native RNA samples were polyadenylated using *E. coli* poly­(A) polymerase (New England Biolabs) and purified using the Oligo Clean & Concentrator Kit (Zymo Research). Direct RNA sequencing libraries were prepared from 1 μg RNA using the SQK-RNA004 kit (Oxford Nanopore Technologies). Yeast samples were multiplexed and demultiplexed using SeqTagger barcodes.^65^ Libraries were sequenced on ONT MinION or PromethION flow cells for up to 72 h using MinKNOW (v24.02.26). Raw signal data were stored in POD5 format.

### 4.3 Neural Network Framework and Calibration Strategy (ModiCal)

RNA modification detection was performed using ModiDeC, a dual-input neural network integrating structured inception blocks and long short-term memory (LSTM) layers for RNA-modification classification.^42^ Data curation, model training, and analysis were carried out using the epi2me-laboratories ModiDeC workflow (v5.2.5).

Building on this framework, we implemented a calibration-driven usage mode, termed ModiCal, to iteratively refine the model for the site-specific validation. Calibration proceeds through baseline training on balanced modified and unmodified synthetic constructs, bulk false-positive suppression using unmodified signal chunks curated from IVT RNA, and iterative single-site calibration targeting residual FPs. A uniform 2% modification threshold was applied during training and evaluation to ensure consistent suppression of background signal across data sets. Higher thresholds were used exclusively for visualization of high-stoichiometry sites.

The full calibration logic and all curation and training parameters are provided in the Supporting Information and summarized schematically in Scheme S1.

## Supplementary Material



## Data Availability

ModiDeC data-curation, training, and analysis workflows used in this study are publicly available at: https://github.com/stegiopast/wf-modidec-data-curation, https://github.com/stegiopast/wf-modidec-training, https://github.com/stegiopast/wf-modidec-analysis. All sequencing data generated in this study have been deposited in the European Nucleotide Archive (ENA) under accession number PRJEB106093. The trained ModiDeC models supporting this study are available in Zenodo (https://doi.org/10.5281/zenodo.18131677).

## Notes

Complete methods; RNA sequences; supplementary schemes; figures; and tables (PDF)
