# Supplementary material for: ModiCal: A Targeted Calibration Workflow for Site-Specific m5C Validation by Nanopore Direct RNA Sequencing
Source: ACS Chem Biol. 2026 May 19;21(6):1291–300. doi: 10.1021/acschembio.6c00009 (PMC13288464; doi:10.1021/acschembio.6c00009)
Supplement: Supplementary file 1 [file cb6c00009_si_001.pdf]

Supporting Information for

ModiCal: A targeted calibration workflow for site-specific m^5C validation by nanopore direct RNA sequencing

Zeynep Özrendeci[1], Stefan Mündnich[1], Stefan Pastore[1], Chia Ching Wu[2], Virginie Marchand[3], Yuri Motorin[3,4], Alessia Ruggieri[2], Susanne Gerber[5,6], Mark Helm[1]**

[1]Institute of Pharmaceutical and Biomedical Science (IPBS), Johannes Gutenberg University, 55128, Mainz, Germany

[2]Department of Infectious Diseases, Molecular Virology, Center for Integrative Infectious Disease Research, Heidelberg University, Medical Faculty Heidelberg, 69120 Heidelberg, Germany

[3]Université de Lorraine, SMP IBSLor, EpiRNA-Seq Core Facility, Nancy F-54000, France

[4]Université de Lorraine, CNRS, UMR7365 IMoPA, Nancy F-54000, France

[5]Institute of Human Genetics, University Medical Center, Johannes Gutenberg University Mainz, 55131 Mainz, Germany

[6]Institute for Quantitative and Computer Biosciences (IQCB), Mainz 55128, Germany

*To whom correspondence should be addressed. E-mail: mhelm@uni-mainz.de, sugerber@uni-mainz.de

Item	Page
Complete Methods	S3
Scheme S1. Overview of the ModiCal calibration workflow	S13
Figure S1. Training-size dependence of baseline model performance.	S14
Figure S2. Iterative single-FP calibration progressively clears residual background in yeast 25S rRNA.	S15
Figure S3. Bisulfite sequencing of m^{5}C sites on *S. cerevisiae* 25S rRNA.	S16
Figure S4. Effect of Dorado probability thresholding on coverage heterogeneity and false-positive m^{5}C calls across yeast 25S rRNA.	S17
Figure S5. Calibration and generalization of the ModiCal m^{5}C model to DENV genomic RNA.	S18
Table S1. Overview of experimental evidence for 25S rRNA m^{5}C at positions C2278 and C2870 in *S. cerevisiae.*	S20

Complete Methods

In vitro transcription of Yeast 25S rRNA.

A circular pUC57 plasmid vector (GenScript, USA) carrying the *S. cerevisiae* 25S rRNA full fragment under the control of a T7 promoter was linearized with BamHI (New England Biolabs, Germany). Enzyme reactions were set up using the recommended buffers and concentrations and incubated overnight at 37 °C. The linearization mixture was purified by sequential organic extractions: two rounds with TE-buffered phenol:chloroform (2:1:1, Carl Roth), followed by two extractions with chloroform and a final wash with diethyl ether to eliminate phenol residues. After each step, the phases were thoroughly mixed and separated by centrifugation. DNA recovery was achieved via ammonium acetate/ethanol precipitation, adding 1/10 volume of 5 M ammonium acetate (Merck-Millipore, Germany), 1 µl glycogen (5 mg/ml, Thermo Fisher Scientific, Germany), and two volumes of absolute ethanol (Carl Roth, Germany). Samples were incubated either for 1 h at −80 °C or overnight at −20 °C. DNA pellets were collected by centrifugation at 12,000 g and −4 °C for 45 min, washed once with 75% cold ethanol, and centrifuged again under the same conditions for 15 min. The dried pellet was resuspended in ultrapure water.

In vitro transcription was then carried out using the HiScribe T7 High Yield RNA Synthesis Kit (New England Biolabs, Germany), following the manufacturer's instructions. The DNA template was removed by digestion with DNase I (2 U per µg DNA; Thermo Fisher Scientific, Germany) at 37 °C for 30 min. The RNA products were purified using the Monarch® RNA Cleanup Kit (New England Biolabs, Germany), and their quality and concentration were assessed by non-denaturing agarose gel electrophoresis and spectrophotometric measurement on a Nanodrop 2000 (Thermo Fisher Scientific, Germany).

RNA oligonucleotide phosphorylation and splint ligation.

For both yeast C2278 and DENV C1218 training constructs, chemically synthesized RNA oligonucleotides were used to generate m^5C modified and unmodified ligation products. For the yeast 25S rRNA, central oligonucleotides carrying an m^5C modification at position 2278 was ordered from Dharmacon (Germany) while an unmodified C2278 carrying central oligonucleotide as well as the flanking unmodified oligonucleotides were purchased from Biomers (Germany). For the DENV use case, analogous oligonucleotides spanning the C1218 site were purchased.

Equimolar mixtures of the three oligonucleotides (5′, central, 3′) were phosphorylated at their 5′ ends using T4 polynucleotide kinase (0.75 U/µl, Thermo Fisher Scientific) per 30 µM RNA in 1× kinase–ligase buffer (50 mM Tris-Cl pH 7.4, 10 mM $MgCl_2$, 5 mM DTT, 2 mM ATP). Reactions were incubated for 1 h at 37 °C. Phosphorylated oligos were then hybridized with a slightly sub-stoichiometric amount (2% less) of complementary DNA splint by heating at 75 °C for 4 min followed by gradual cooling to room temperature over 15 min.

Ligation was initiated by adding T4 DNA ligase (2 U/µl, Thermo Fisher Scientific) and incubating overnight at 16 °C. After ligation, residual DNA splint was degraded with DNase I (0.1 U/µl, Thermo Fisher Scientific) at 37 °C for 30 min. The resulting ligated RNA constructs were purified by electrophoresis on 8% or 10% denaturing polyacrylamide gels. RNA integrity and concentration were determined using a NanoDrop 2000 spectrophotometer (Thermo Fisher Scientific).

Analysis of m^5C modifications by BisulfiteSeq

Total RNA (~50 ng) from *S.cerevisiae* was subjected to bisulfite treatment according to published protocols [16]. RNA was converted to a DNA library using NEBNext Small RNA

Library kit (NEB, UK). The DNA library was quantified using a fluorometer (Qubit 2.0 fluorometer, Invitrogen, USA) and qualified using a High Sensitivity DNA chip on Agilent Bioanalyzer 2100. Libraries were multiplexed and subjected for high-throughput sequencing on an Illumina NextSeq2000 instrument with a 2x50 nt paired-end read mode. BS-Seq analysis of m^5C/m^4C locations was performed using alignments of the sequencing reads to a C to U converted reference sequence, followed by determining the residual C non-deamination. The proportion of residual non-deaminated C (UtoC MM score) corresponds to the molar ratio of m^5C/m^4C at a given location. The UtoC score was calculated by analysis of the match string from full mpileup format.

Analysis of DENV samples by Nanopore DRS.

Generation of the DENV IVT transcripts, purification of the corresponding DENV gRNA samples, and their nanopore direct RNA sequencing were performed as previously described [64].

Polyadenylation and direct RNA sequencing library preparation

The ligated RNA products (m^5C-modified and unmodified) from both yeast C2278 and DENV C1218 constructs, as well as their full-length IVTs and native transcripts (*S. cerevisiae* 25S rRNA from WT, Nop2 KO and Rcm1 KO strains; DENV WT gRNA), were 3′-polyadenylated using E. coli poly(A) polymerase (New England Biolabs) according to the manufacturer's instructions. Polyadenylated RNAs were purified with the Oligo Clean & Concentrator Kit (Zymo Research) and eluted in nuclease-free water prior to library construction. Direct RNA sequencing libraries were prepared from 1 µg of polyadenylated RNA using the SQK-RNA004 kit (Oxford Nanopore Technologies) following the manufacturer's protocol. Where indicated, yeast samples were multiplexed using SeqTagger barcodes and demultiplexed with the SeqTagger tool [67]. Final

libraries were quantified using the Qubit dsDNA HS Assay Kit (Invitrogen), loaded onto ONT MinION and PromethION R10.4.1 flow cells, and sequenced for up to 72 h under MinKNOW software (v24.02.26). Raw signal data were recorded in POD5 format and used as input for all downstream bioinformatic and ModiCal analyses.

ModiCal pipeline

We implemented our calibration workflow on top of ModiDeC, a dual-input neural network (~20 M parameters) that combines structured inception blocks and long short-term memory (LSTM) layers for RNA-modification classification. All data curation, training and analysis steps were carried out using the epi2me-labs implementation of ModiDeC, which provides reproducible workflows for training modification classifiers on synthetic RNA strands and applying them to native transcripts of interest. In ModiCal, these workflows are organized into three stages: (i) baseline training on synthetic constructs, (ii) bulk false-positive (FP) suppression using IVT-derived unmodified signals, and (iii) iterative single-FP calibration. A detailed step-by-step protocol including exact workflow settings and helper scripts is provided in Supplementary Fig. S1 and in the accompanying GitHub repository.

1. Baseline curation and training

For the yeast use case, we focused on the well-characterized m^5C2278 site in 25S rRNA; for the DENV use case, we targeted the candidate m^5C1218 site in the viral genomic RNA. In both cases, baseline training was performed on short splint-ligated synthetic RNAs that mimic the local sequence context and carry either a methylated or unmodified cytidine at the target position.

Baseline data curation was performed with the ModiDeC Curation workflow on POD5–BAM–FASTA triplets for modified and unmodified ligation constructs. Unless stated otherwise, the following settings were used: flow cell type RNA004; curation type "Training"; Direct RNA

enabled; modification mapping enabled to generate chunks centered on the target cytidine; modification dictionary/type set to m^5C. For modified constructs, the "Modified data" flag was set to "yes"; for unmodified constructs, it was set to "no". The "Location of modification" field was set to the local position of the target cytidine within the splint construct. For each class, training entities were stored at 512 chunks per output .npz file, and read indices were initially sampled from 0 to 100,000, yielding at least 1,000 .npz files per class. If more files were produced, custom shell scripts were used to shuffle and randomly subsample 800 .npz files per class for training and 200 .npz files per class for validation.

The resulting 800 modified and 800 unmodified .npz files were pooled into a single training set (1,600 files total), and the 200 + 200 .npz files into a validation set (400 files total). These pools were used as input to the ModiDeC Training workflow with a batch size of 128, a k-mer model of 9 and four training epochs, resulting in the baseline model.

Baseline analysis on native RNA was then performed with the ModiDeC Analysis workflow using POD5 files containing raw signal and the corresponding aligned BAM files, together with the baseline model and the appropriate reference FASTA (yeast 25S rRNA or DENV2 genomic RNA). We used the RNA004 configuration, a chunk size of 400 samples, a maximum sequence length of 40, a processing batch size of 512 and 32 CPU threads, with the modification dictionary restricted to m^5C. For each reference position, per-site modification frequency was computed as the number of reads classified as "modified" divided by the coverage at that position. Apparent FP sites were defined as positions with m^5C frequency above a conservative cut-off (typically 2%) and were exported using a custom Python script (extract_fp_sites.py) that reports both the list of genomic positions and a matching list of m^5C labels for downstream curation.

2. Bulk false-positive (FP) curation and training

To generate unmodified reference data for all FP sites in a single step, we curated signal chunks from full-length IVT RNA corresponding to each FP coordinate identified in the baseline analysis. IVT curation settings mirrored those used for the ligation constructs, except that the "Modified data" flag was set to "no" and both the "Location of modification" and "Modification type" fields contained space-separated lists derived from the extract_fp_sites.py output (FP positions and corresponding m^5C labels). The flow cell type was set to RNA004, curation type to "Training", modification mapping to "yes", and 512 entities were stored per .npz file. Read indices were sampled from 0 to 5,000, and the upper bound was increased as needed to obtain at least 600 .npz files from IVT. If more than 600 files were generated, a helper script was used to shuffle and randomly select 600 IVT .npz files for inclusion in the training pool.

These 600 IVT-derived .npz files were added to the existing ligation-based training set, resulting in a ratio of modified ligation : unmodified ligation : IVT = 800 : 800 : 600, while the validation set (200 + 200 ligation files) was kept unchanged. Training was repeated with the same hyperparameters (batch size 128, k-mer 9, four epochs), yielding the "BulkFP" model. This model was then applied to the same native RNA datasets with identical Analysis settings as for the baseline model, and an updated list of FP sites above the 2% threshold was obtained. Maintaining the same internal calling threshold across calibration steps ensured that background levels could be compared directly between models and across independent sequencing runs and yeast strains. For final visualization, display thresholds were adjusted to match the expected stoichiometry of the true-positive site (5% for highly modified positions such as yeast C2278 and 2% for low-stoichiometry sites such as DENV C1218).

3. Iterative single-FP curation and training

Residual FP sites after the BulkFP step were resolved by iterative single-site calibration. For each remaining FP coordinate, unmodified signal chunks were curated from full-length IVT RNA using the same Curation settings as for the bulk IVT step, but specifying a single target position in the “Location of modification” field and restricting the “Modification type” to m^5C. To ensure sufficient sampling for each site, the read index range was extended to 0–10,000, typically yielding at least ~10 .npz files per FP site. To keep the global training set balanced while still providing site-specific negative examples, only one .npz file per FP site was added to the training pool in each calibration round.

In the first calibration round (Cal1), a single .npz file for each residual FP site was appended to the existing training pool (ligation constructs + bulk IVT). The model was retrained with unchanged hyperparameters and re-applied to the same native RNA reads. FP sites that dropped below the 2% threshold were considered resolved and were not targeted further, whereas persistent or newly emerging FPs were included in the next round by adding one additional .npz per site. This procedure (Cal2, Cal3, …) was repeated until no cytidine in the reference RNA exceeded the FP threshold. The final calibration model for each use case (yeast 25S rRNA C2278 and DENV gRNA C1218) was then used for all downstream analyses, including wild-type/knockout comparisons and benchmarking against Dorado.

Sequences for the coding strand of insert DNA used for the synthesis of RNA

The bold sequences are the T7 promoter on the 5` end and the poly-A tail for library preparation

on the 3` end.

25S rRNA

5′-taatacgactcactataggg

tttgacctcaaatcaggtaggagtacccgctgaacttaagcatatcaataagcggaggaaaagaaaccaaccgggattgccttagtaacg

gcgagtgaagcggcaaaagctcaaatttgaaatctggtaccttcggtgcccgagttgtaatttggagagggcaactttggggccgttccttgt

ctatgttccttggaacaggacgtcatagagggtgagaatcccgtgtggcgaggagtgcggttctttgtaaagtgccttcgaagagtcgagttg

tttgggaatgcagctctaagtgggtggtaaattccatctaaagctaaatattggcgagagaccgatagcgaacaagtacagtgatggaaagat

gaaaagaactttgaaaagagagtgaaaaagtacgtgaaattgttgaaagggaagggcatttgatcagacatggtgttttgtgccctctgctcct

tgtgggtaggggaatctcgcatttcactgggccagcatcagttttggtggcaggataaatccataggaatgtagcttgcctcggtaagtattata

gcctgtgggaatactgccagctgggactgaggactgcgacgtaagtcaaggatgctggcataatggttatatgccgcccgtcttgaaacacg

gaccaaggagtctaacgtctatgcgagtgtttgggtgtaaaacccatacgcgtaatgaaagtgaacgtaggttggggcctcgcaagaggtg

cacaatcgaccgatcctgatgtcttcggatggatttgagtaagagcatagctgttgggacccgaaagatggtgaactatgcctgaatagggtg

aagccagaggaaactctggtggaggctcgtagcggttctgacgtgcaaatcgatcgtcgaatttgggtataggggcgaaagactaatcgaa

ccatctagtagctggttcctgccgaagtttccctcaggatagcagaagctcgtatcagttttatgaggtaaagcgaatgattagaggttccggg

gtcgaaatgaccttgacctattctcaaactttaaatatgtaagaagtccttgttacttaattgaacgtggacatttgaatgaagagcttttagtgggc

catttttggtaagcagaactggcgatgcgggatgaaccgaacgtagagttaaggtgccggaatacacgctcatcagacaccacaaaaggtg

ttagttcatctagacagccggacggtggccatggaagtcggaatccgctaaggagtgtgtaacaactcaccggccgaatgaactagccctg

aaaatggatggcgctcaagcgtgttacctatactctaccgtcagggttgatatgatgccctgacgagtaggcaggcgtggaggtcagtgacg

aagcctagaccgtaaggtcgggtcgaacggcctctagtgcagatcttggtggtagtagcaaatattcaaatgagaactttgaagactgaagtg

gggaaaggttccacgtcaacagcagttggacgtgggttagtcgatcctaagagatggggaagctccgtttcaaaggcctgattttatgcagg

ccaccatcgaaagggaatccggttaagattccggaacctggatatggattcttcacggtaacgtaactgaatgtggagacgtcggcgcgag

ccctgggaggagttatcttttcttcttaacagcttatcaccccggaattggtttatccggagatggggtcttatggctggaagaggccagcacct

ttgctggctccggtgcgcttgtgacggcccgtgaaaatccacaggaaggaatagttttcatgccaggtcgtactgataaccgcagcaggtctc

caaggtgaacagcctctagttgatagaataatgtagataagggaagtcggcaaaatagatccgtaacttcgggataaggattggctctaagg

gtcgggtagtgagggccttggtcagacgcagcgggcgtgcttgtggactgcttggtggggcttgctctgctaggcggactacttgcgtgcct

tgttgtagacggccttggtaggtctcttgtagaccgtcgcttgctacaattaacgatcaacttagaactggtacggacaaggggaatctgactgt

ctaattaaaacatagcattgcgatggtcagaaagtgatgttgacgcaatgtgatttctgcccagtgctctgaatgtcaaagtgaagaaattcaac

caagcgcgggtaaacggcgggagtaactatgactctcttaaggtagccaaatgcctcgtcatctaattagtgacgcgcatgaatggattaac

gagattcccactgtccctatctactatctagcgaaaccacagccaagggaacgggcttggcagaatcagcggggaaagaagaccctgttga

gcttgactctagtttgacattgtgaagagacatagagggtgtagaataagtgggagcttcggcgccagtgaaataccactacctttatagtttct

ttacttattcaatgaagcggagctggaattcattttccacgttctagcattcaaggtcccattcggggctgatccgggttgaagacattgtcaggt

ggggagtttggctggggcggcacatctgttaaacgataacgcagatgtcctaagggggctcatggagaacagaaatctccagtagaaca

aaagggtaaaagccccttgattttgattttcagtgtgaatacaaaccatgaaagtgtggcctatcgatcctttagtccctcggaatttgaggcta

gaggtgccagaaaagttaccacagggataactggcttgtggcagtcaagcgttcatagcgacattgctttttgattcttcgatgtcggctcttcc

tatcataccgaagcagaattcggtaagcgttggattgttcacccactaatagggaacgtgagctgggtttagaccgtcgtgagacaggttagtt

ttaccctactgatgaatgttaccgcaatagtaattgaacttagtacgagaggaacagttcattcggataattggtttttgcggctgtctgatcagg

cattgccgcgaagctaccatccgctggattatggctgaacgcctctaagtcagaatccatgctagaacgcggtgatttctttgctccacacaat

atagatggatacgaataaggcgtccttgtggcgtcgctgaaccatagcaggctagcaacggtgcacttggcggaaaggccttgggtgcttg

ctggcgaattgcaatgtcattttgcgtggggataaatcatttgtatacgacttagatgtacaacggggtattgtaagcagtagagtagccttgttg

ttacgatctgctgagattaagcctttgttgtctgatttgtttgaccctctctcagaaacaaagtgtacgttgaaataaggtacgggaggtacttgga

gcggccgcaaaaaaaaaaaaaaaaaaaaaaaaaaaaaaaaaaaaaaaaaaaaaaaaaaaaaaagccgaattcttaattggatcctggagctcc

agcttttgtt

Oligoribonucleotide sequences for the splint ligated modified and unmodified ground truth synthesis

Oligonucleotide Name	Full Sequence (5’-3’)
***S.cerevisiae* 25S rRNA C2278 Ground Truth (Left Arm)**	ACCAAGCGCGGGUAAACGGCGGGAGUAACUAUGACUCUCU

S.cerevisiae **25S rRNA C2278 Ground Truth (Right Arm)**	GUCAUCUAAUUAGUGACGCGCAUGAAUGGAUUAACGAGAU
S.cerevisiae **25S rRNA C2278 Ground Truth (Middle, modified)**	UAAGGUAGC(m^5C)AAAUGCCUC
S.cerevisiae **25S rRNA C2278 Ground Truth (Middle, unmodified)**	UAAGGUAGCCAAAUGCCUC
S.cerevisiae **25S rRNA C2278 Ground Truth (Splint cDNA)**	ATCCATTCATGCGCGTCACTAATTAGATGACGAGGCATTTGGCTACCTTAAGAGAGTCATAGTTACTCCCGCCGTTTACCCG
**DENV gRNA C1218 Ground Truth (Left Arm)**	GAACCCAGCCUAAAUGAAGAGCAGGACAAAAGGUUCGUCU
**DENV gRNA C1218 Ground Truth (Right Arm)**	CAGAGGAUGGGGAAAUGGAUGUGGAUUAUUUGGAAAAGGA
**DENV gRNA C1218 Ground Truth (Middle, modified)**	GCAAACA(m^5C)UCCAUGGUGGA
**DENV gRNA C1218 Ground Truth (Middle, unmodified)**	GCAAACACUCCAUGGUGGA
**DENV gRNA C1218 Ground Truth (Splint cDNA)**	AAATAATCCACATCCATTTCCCCATCCTCTGTCCACCATGGAGTGTTTGCAGACGAACCTTTTGTCCTGCTCTTCATTTAGG

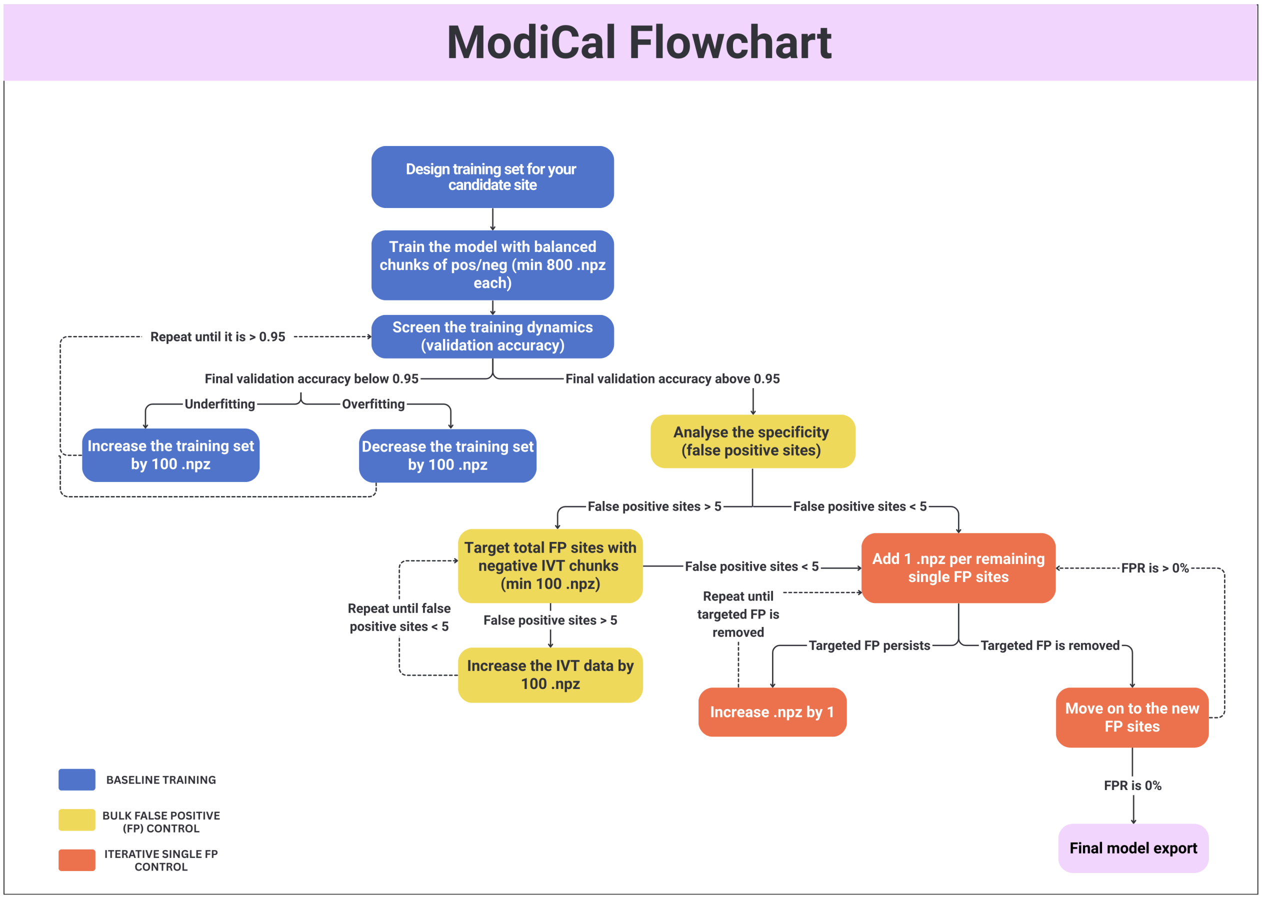

Scheme S1. Flowchart summarizing the ModiDeC-based training-calibration (ModiCal) workflow. The pipeline comprises three stages: baseline training (blue), bulk false-positive (FP) control (yellow), and iterative single-FP correction (orange). Model performance is first assessed by validation accuracy; once accuracy exceeds 0.95, specificity is evaluated by the number of FP sites and the false-positive rate (FPR). Training set size is adjusted in ±100 .npz increments to avoid under- or overfitting. If >5 FP sites are detected, bulk FP control retrains the model with additional unmodified IVT chunks (≥100 .npz) until the FP count falls below five. Remaining FP sites are then targeted individually by incremental addition of negative chunks (+1 .npz per iteration) until FPR = 0, after which the final calibrated model is exported. This rule-based decision tree formalizes the calibration logic into a reproducible framework for high-precision RNA modification detection.

A.

B.

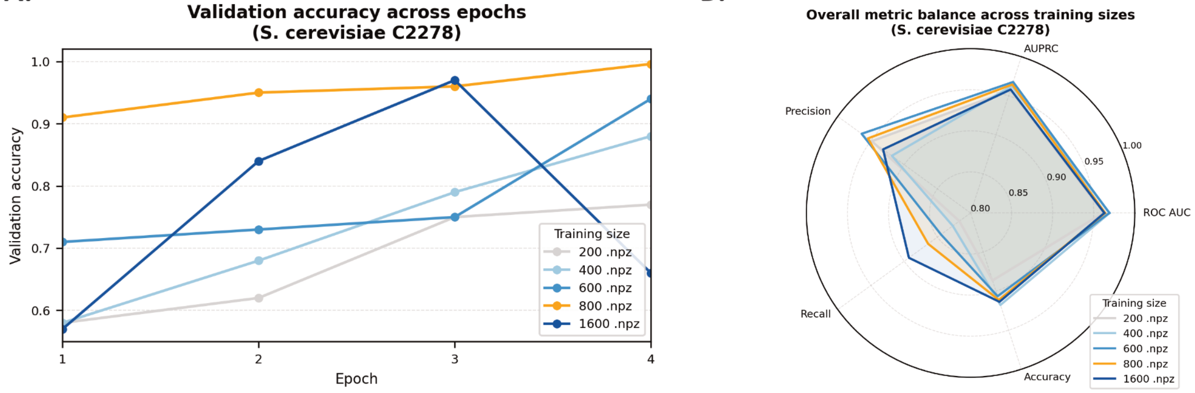

Figure S1. Training-size dependence of baseline model performance. (A) Validation accuracy across four training epochs for baseline models trained with 200–1600 .npz files per class. (B) Radar plot summarizing ROC–AUC, AUPRC, accuracy, precision, and recall for each training size.

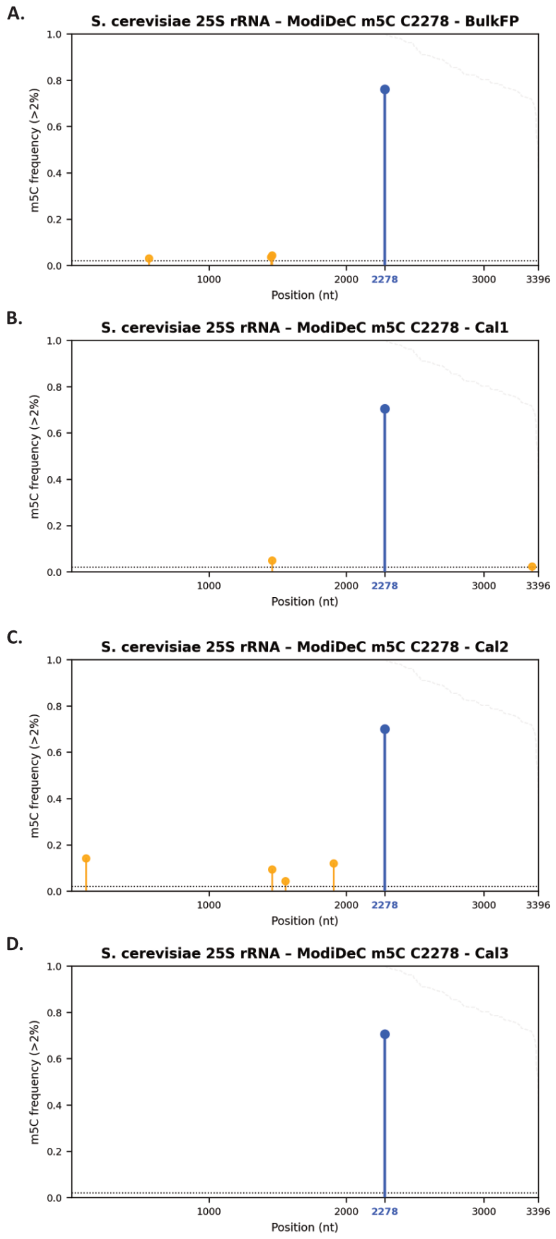

Figure S2. Iterative single-FP calibration progressively clears residual background in yeast 25S rRNA. (A–D) m^5C frequency profiles across *S. cerevisiae* 25S rRNA after BulkFP (A) and successive calibration rounds Cal1 (B), Cal2 (C), and Cal3 (D) using the ModiDeC m^5C model. The predicted m^5C site at C2278 is indicated in blue, while residual false-positive sites are shown in orange. The dashed horizontal line denotes the 2% calling threshold. Gray traces indicate read coverage.

A.

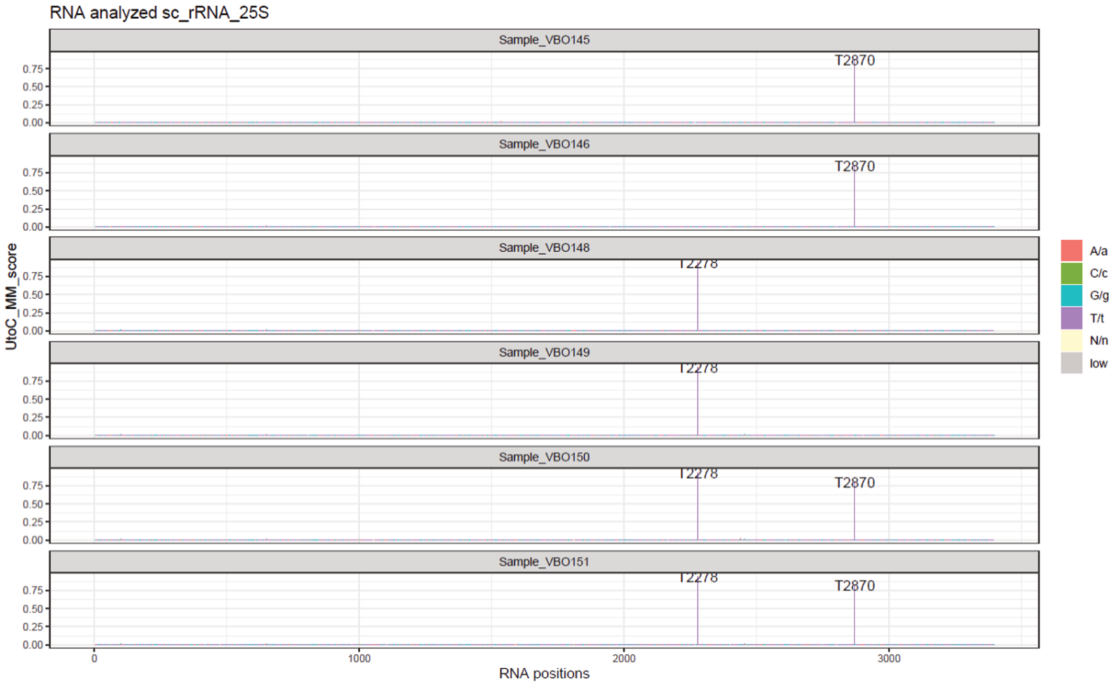

B.

Sample	Aligned reads	Strain genotype	Referred as
**VBO145**	12,504,176	ΔYNL022c mat Alpha	Rcm1 knockout, Replicate 1
**VBO146**	14,873,434	ΔYNL022c mat A	Rcm1 knockout, Replicate 2
**VBO148**	14,459,538	ΔYNL061 + Nop2 DB_1B n°1	Nop2 knockout, Replicate 1
**VBO149**	14,451,874	ΔYNL061 + Nop2 DB_1C	Nop2 knockout, Replicate 2
**VBO150**	15,911,927	ΔYNL061 + Nop2 DB_1N n°3	Wild Type, Replicate 1
**VBO151**	15,842,563	ΔYNL061 + Nop2 DB_2C	Wild Type, Replicate 2

Figure S3. Bisulfite sequencing of m^{5}C sites on *S. cerevisiae* 25S rRNA. (A) UtoC_MM score profiles along the yeast 25S rRNA for Rcm1 knockout, Nop2 knockout, and wild-type samples. rRNA modification mapping was done using BSSeq protocol, potentially detecting bisulfite-resistant m^{5}C / m^{4}C RNA residues. The BSSeq signal shows residual non-deaminated C content in RNA (UtoC_MM_score) (see Methods). RNA sequence and numbering of positions are shown at the bottom. Color code corresponds to the nature of nucleotide in RNA. Vertical lines indicate positions C2278 and C2870. (B) Overview of samples, aligned read counts, strain genotypes, and experimental grouping used in this analysis.

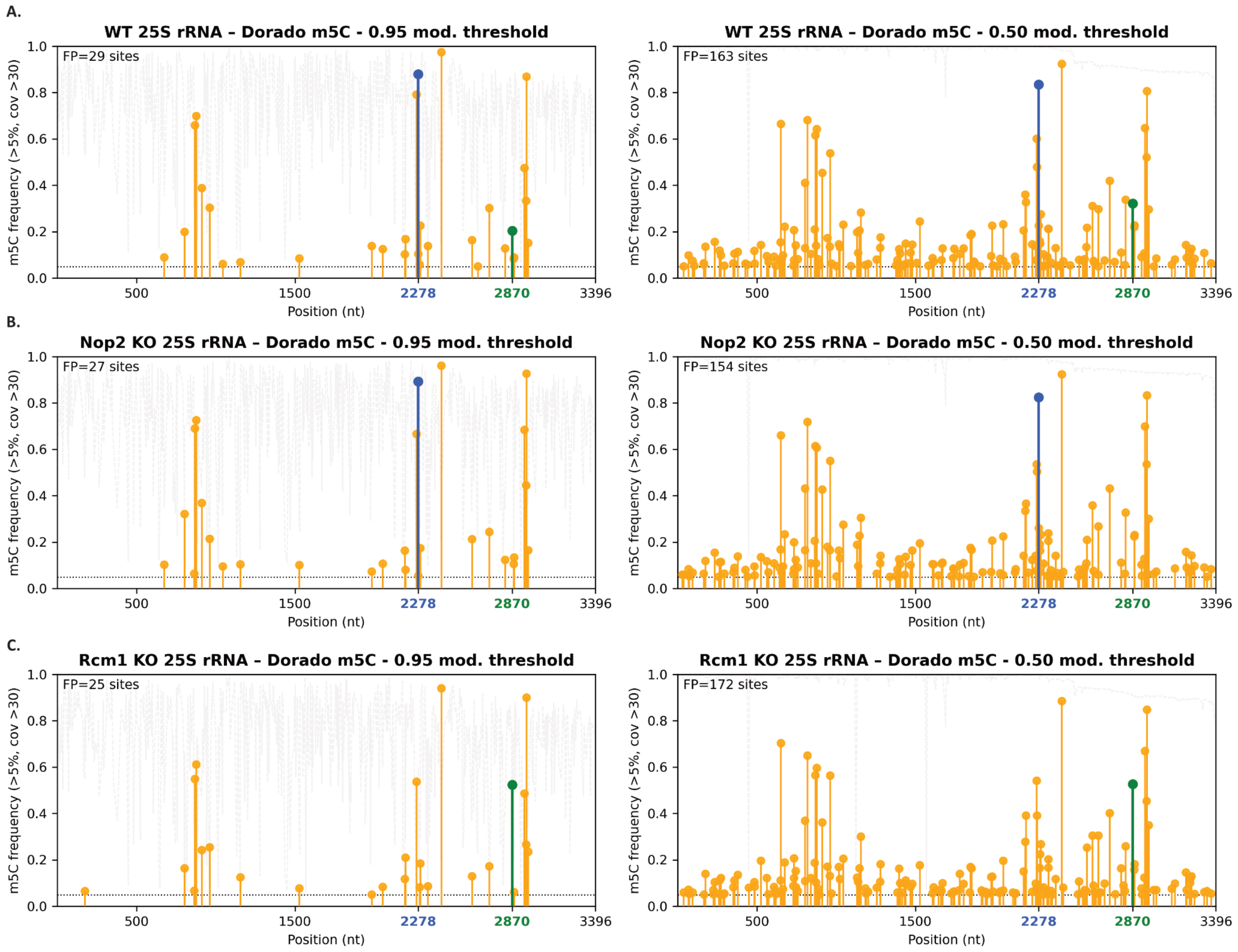

Figure S4. Effect of Dorado probability thresholding on coverage heterogeneity and false-positive m^{5}C calls across yeast 25S rRNA. Dorado m^{5}C profiles for WT (A), nop2Δ (B), and rcm1Δ (C) at probability cutoffs 0.95 (left) and 0.50 (right). Gray trace: per-position coverage. C2278 (blue) and C2870 (green) are highlighted; off-target calls are orange. Lowering the cutoff increases coverage continuity but inflates FP calls (counts shown).

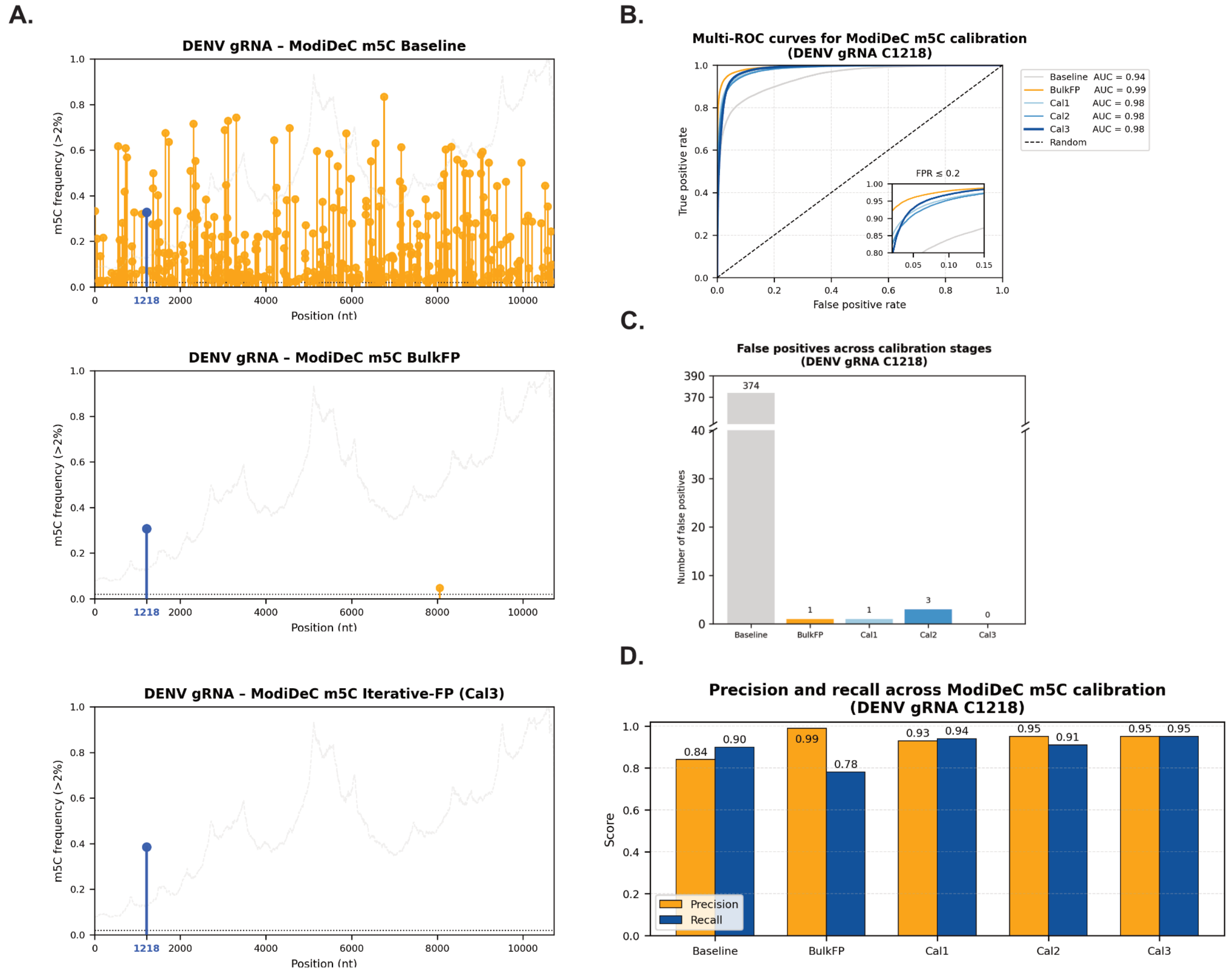

Figure S5. Calibration and generalization of the ModiCal m^{5}C model to DENV genomic RNA. 99-nt synthetic constructs mimicking the local DENV sequence context around C1218 and carrying either m^{5}C or canonical C at the central position were used as ground truth for baseline training, while a separate IVT DENV RNA served as the unmodified reference for BulkFP and iterative single-FP calibration. Given the low stoichiometry of m^{5}C1218 (≈15–25% by bisulfite sequencing), a uniform 2% threshold was applied to retain genuine low-level signal. **(A)** m^{5}C profiles across DENV gRNA evolving through ModiCal steps. The baseline model (top) correctly detected C1218 at ~33% but produced 374 additional positions above the 2% threshold, including

several FPs above 80%. A single BulkFP retraining round (middle) collapsed this background from 374 to one residual site while preserving the C1218 signal (~31%). Three iterative single-FP calibration rounds (Cal1–Cal3; bottom) then eliminated the remaining background, with m^5C1218 stably detected at 29–33% throughout and all other positions below 2%. **(B)** Multi-ROC analysis; discrimination improved from AUC ~0.94 at baseline to ~0.99 after BulkFP and stabilised at ~0.98 across Cal1–Cal3. **(C)** False-positive counts across calibration stages. **(D)** Precision–recall across rounds, showing a shift from over-calling at baseline to a balanced Cal3 state (precision/recall both ~0.95). Together these data establish that the ModiCal workflow developed on yeast rRNA generalises to a structurally and biologically distinct RNA, converting a broadly trained synthetic-construct model into a high-precision detector that reports a single, low-stoichiometry m^5C peak at C1218 with no detectable background elsewhere along the ~10 kb viral genome.

Year	Reference	m^5C2278 (WT)	m^5C2870 (WT)	Main method / readout
1973	Klootwijk & Planta [59]	Not site-resolved; total of two m^5C per 25S rRNA molecule	Not site-resolved; total of two m^5C per 25S rRNA molecule	2D paper chromatography / electrophoresis of nucleosides
2013	Sharma et al. [53]	Near-stoichiometric in WT; absent in rcm1Δ	Near-stoichiometric in WT; absent in nop2Δ	RP-HPLC (global), bisulfite sequencing (site-specific)
2014	Gigova et al. [54]	Near-stoichiometric in WT; absent in rcm1Δ	Not analysed	RP-HPLC; bisulfite sequencing + mutational analysis
2015	Schosserer et al. [52]	≥80% in WT; absent in rcm1Δ	Not analysed	Barcoded bisulfite sequencing (IonTorrent)
2015	Bourgeois et al. [55]	Near-stoichiometric; absent in rcm1Δ	Near-stoichiometric; absent in nop2Δ	HPLC–MS/MS; bisulfite sequencing

Table S1. Overview of experimental evidence for 25S rRNA m^5C at positions C2278 and C2870 in *S. cerevisiae.*
